# Polycystic Ovary Syndrome (PCOS) and Non-Suicidal Self-Injury (NSSI): A Community-Based Study

**DOI:** 10.3390/healthcare10061118

**Published:** 2022-06-15

**Authors:** Sophie Williams, Dean Fido, David Sheffield

**Affiliations:** School of Psychology, University of Derby, Kedleston Road, Derby DE22 1GB, UK; d.fido@derby.ac.uk (D.F.); d.sheffield@derby.ac.uk (D.S.)

**Keywords:** Polycystic Ovary Syndrome (PCOS), suicidal ideation, non-suicidal self-injury (NSSI), emotion regulation

## Abstract

Polycystic Ovary Syndrome (PCOS) is an endocrine condition that has been associated with atypical emotional regulation strategy use as well as elevated levels of depression, anxiety, self-harm and suicidal ideation. Despite the existence of clinical screening guidance for this population, there is still little to no understanding of how non-suicidal self-injury and suicidal ideation and intention manifest in women with PCOS and how this might differ from women without PCOS. Within this cross-sectional investigation, women with and without a diagnosis of PCOS (*n* = 418) completed validated metrics of emotion dysregulation, rumination and non-suicidal self-injury (NSSI), as well as self-reported indices of previous suicidal ideation and future suicidal intention. Group comparisons indicated that women with, relative to those without, PCOS reported significantly greater metrics across all variables. Moreover, serial mediation analyses were conducted to test the ideation-to-action framework of suicide in women with PCOS, with the positive relationship between a PCOS diagnosis and future suicidal intention being explained through the indirect pathway of increased emotion dysregulation, recent suicidal ideation and NSSI. Our findings call to action the need for international screening for suicide intention and self-harm in women with PCOS.

## 1. Introduction

Polycystic Ovary Syndrome (PCOS) is the most common endocrine condition globally [1], impacting approximately 8–13% of women of reproductive age [2,3]. PCOS is the main cause of anovulatory infertility [2] with common symptoms including acne, amenorrhea or oligomenorrhea, hirsutism, obesity, hyperandrogenism and polycystic ovaries [1,2]. 

Knowledge of personality characteristics of women with PCOS is limited [4,5]; however, initial research suggests that women with PCOS may experience dysfunctional emotional regulation [4,5]. Specifically, Marsh et al. detail that insulin-resistant women with PCOS demonstrate differences during emotional processing in the prefrontal cortex in comparison to those without PCOS, suggesting a potential underlying biological difference impacting emotion regulation between women with and without PCOS. However, there is little research exploring the relationship between emotion regulation and mood disorders in PCOS [4].

It is well known that women with, relative to without, PCOS report higher levels of depression and anxiety in clinical [6,7,8,9,10] and community-based samples [11]. Recently, these data led to international recommendations for women with PCOS to be screened for anxiety and depression [7,10], resulting in a clinical consensus recommendation by the 2018 European Society of Human Reproduction and Embryology (ESHRE) to call for the screening of depression and anxiety in women newly diagnosed with PCOS [12]. 

It is unsurprising then that PCOS has been associated with an increased risk of hospital admissions for stress, anxiety, depression and self-harm [13]. Qualitative data also indicate that women with PCOS may engage in self-harm behaviours and suicidal ideation and may perceive these behaviours as linked with their PCOS [14]. Indeed, anovulatory infertility that is associated with PCOS has been previously linked to increased suicidal risk [15,16]. Furthermore, there is limited but initial evidence of suicide ideation in women with PCOS [14,17]. Notably, Månsson et al. [18] identified that suicide attempts were seven times more common for women with PCOS compared to age-matched controls [18]. More recently, a nationwide Swedish cohort study identified that women with PCOS were 40% more likely to attempt suicide than other women [19]. However, as deliberate self-harm is the strongest risk factor for future suicide [20], obtaining a greater understanding of suicidal ideation and self-harm behaviours in PCOS is essential to safeguard and provide suitable interventions in this population. 

One potential means of examining suicidal intent is through the ideation-to-action framework [21], which aids our understanding as to why only a proportion of those who engage in suicide ideation consider attempting suicide. Specifically, both intention and capability must be independently present [22] as one evidences the capability to overcome fear involved in the contemplation, planning, preparation and action involved in suicide attempts [23]. Most recently, Fadoir et al. [24] examined this framework in the context of psychopathic personality traits, where individuals with heightened scores of secondary psychopathy (indicative of antisocial behaviour and disinhibition; [25]) were associated with more prevalent suicidal intention through the mechanism of increased emotion dysregulation, rumination, past suicidal ideation and non-suicidal self-injury (NSSI) [24]. Theory pertaining to this pathway is described below.

Rumination is a maladaptive emotion regulation strategy whereby one repetitively engages in negative thoughts pertaining to extreme affective situations, which in turn prevents effective emotion regulation and the attenuation of depressive symptoms that might underpin suicidal ideation [26,27]. On a neurocognitive level, disruption to brain networks that enable effective top-down control of emotive responses have been identified in females who use rumination strategies during stress-inducing situations [28]. Emotion dysregulation in conjunction with rumination might also lead to a greater propensity to engage in NSSI, whereby NSSI forms either a physical strategy of reducing extreme emotions [29] or as a means of distraction, self-punishment, or indication of a need for help [30]. In turn, NSSI is associated with increased suicidal intention and attempts in the future [31], potentially and in relation to the ideation-to-action framework by providing evidence of one’s capability to engage in self harm and suicide-related behaviour and attenuation to the fear and pain associated with it [32]. Taken together, with women with PCOS known to experience emotion dysregulation [4], the ideation-to-action framework described above provides a logical means of exploring the mechanisms underpinning increased suicidal intent within this population [18]. 

To our knowledge, this is the first research to investigate how dysregulated emotions, rumination, recent suicidal ideation and NSSI predict future suicidal intention in women with PCOS. Using a serial mediation model, we hypothesized that the presence of a diagnosis of PCOS would not only be positively associated with all components of and precursors to suicidal ideation and intent, but that the relationship between having PCOS and future suicidal intent would be explained (mediated) through the indirect effects of emotion dysregulation, rumination, recent suicidal ideation and NSSI. Moreover, differences in these measures will be examined between women with and without PCOS, where it is expected that women with PCOS will exhibit higher scores and greater prevalence on average.

## 2. Materials and Methods

### 2.1. Participants

To ensure that our data were robust enough to hold practical importance, we sought to recruit at least 400 participants, which is similar to that of research exploring sexual health behaviour (*n* = 358; [33]) and twice that of recent research exploring suicidal ideation (*n* = 201; [24]) using comparable analysis pathways. After removing cases where more than 5% of the data were missing (*n* = 3), a total of 429 UK participants (M_age_ = 32.91 years, SD = 10.31, 27% PCOS+, range = 18–70) had completed an online questionnaire, which was advertised through professional social networks belonging to the researchers, and further supplemented through the crowdsourcing website Prolific. Prolific is considered a viable means of participant recruitment, with data quality comparable to that obtained through face-to-face means [34]. Inclusion criteria were that participants had to be fluent in English and from the UK (to ensure congruency in healthcare provision), aged 18 years or older, and without personality-based mental health conditions (such as bipolar disorder, schizophrenia, or any condition that has required hospitalisation over the last 12 months). All crowdsourced completers were reimbursed with £1.20 for their participation.

### 2.2. Materials

#### 2.2.1. Demographics

Participants were asked to report their age, level of education, ethnicity and whether they had received a formal diagnosis of PCOS. Specifically, education was measured ordinally under the categories: School (normally up to the age 16), College (normally up to the age 18), University (Bachelor’s degree or equivalent), University (Master’s degree) and University (Doctorate or PhD). 

#### 2.2.2. Difficulties in Emotion Regulation Scale (DERS)

The DERS [35] is a 36-item self-report measure of clinical difficulties in emotion regulation (e.g., “I am confused about how I feel”) scored using a 5-point scale from 1 (“almost never”) to 5 (“almost always”). In this study, the total score was used, with high scores indicative of greater emotion regulation difficulties. 

#### 2.2.3. Ruminative Responses Scale (RRS)

The RRS [36] is a 22-item self-report frequency measure of one’s propensity to ruminate (e.g., “Think about how alone you feel”) scored using a 4-point scale from 1 (“almost never”) to 4 (“almost always”). High scores indicated more prevalent rumination.

#### 2.2.4. Deliberate Self-Harm Inventory Self-Injurious (DSHI) 

The DSHI [37] is a 17-item self-report measure of destructive body tissue-related behaviour without conscious suicidal intent. Participants were asked to respond to 17 behaviours (including one which they could add themselves) as to whether they had “… ever intentionally (i.e., on purpose)…” engaged in those behaviours. Responses of “Yes” were scored as 1, and responses of “No” were scored as 0. Total scores were generated for each participant with higher scores indicative of greater self-harm behaviour use. 

#### 2.2.5. Suicide Behaviors Questionnaire-Revised (SBQ-R)

The SBQ-R [38] is a 4-item self-report measure of self-injurious thoughts and behaviour. In this study, only two items were measures. Item 2 asked (“How often have you thought about killing yourself in the past year?”) and was scored from 0 (“Never”) to 4 (“Very Often [5 or more times]”), with higher scores indicating greater recent (i.e., over a 12-month period) suicidal ideation. Item 4 asked, “How likely is it that you will attempt suicide someday?” and was scored from 0 (“Never”) to 6 (“Very Likely”), with higher scores indicating greater future suicidal intention. 

### 2.3. Procedure

The research was approved by an institutional ethical review panel prior to data collection. Initially, participants gave consent in accordance with approved central university research protocols and national ethical guideline and then entered their demographic information in Qualtrics survey software. Following this, DERS, RRS, DSHI and SBQ-R were presented in a randomized order. Randomization was conducted for each participant by the survey software to reduce the likelihood of order effects influencing the data. Participants were debriefed and re-affirmed their consent by ticking a box on the last pages of our online survey. On average, the study took less than 15 min to complete. 

### 2.4. Analysis

Pearson correlations were computed between the focal predictor (diagnosis of PCOS), the dependent variable (future suicidal ideation) and the mediator variables (emotional dysregulation, rumination, recent suicidal ideation, NSSI) for the whole sample. Then, we computed *t*-tests to compare variables of interest between women with and without PCOS. Finally, we used Model 6 of the PROCESS plugin for SPSS (v3.4; [39]) to run a serial mediation model with the covariates of age, education and ethnicity.

## 3. Results

### 3.1. Preliminary Analyses

Eleven participants (three with PCOS) were excluded due to their data breaching two or more cut-offs for measures of Mahalanobis, Cook’s distance and leverage using a Chi squared value of 26.13 (*p* < 0.001), leaving a final sample of 418 (M_age_ = 32.93 years, SD = 10.32, 27% PCOS+, 86.4% White British, 5% African–Caribbean). There were no missing data points in the final sample. 

### 3.2. Prevalence

To assess differences between participants with and without a diagnosis of PCOS, independent-samples *t*-tests were computed. In our sample, women with PCOS were typically younger and less educated than women without PCOS, with small to medium effect sizes. Specifically, women with PCOS typically had education at college (39.8%), university (31.9%) and school (13.3%) levels, with women without PCOS typically having education at university (44.6%), college (29.2%) and Master’s (15.7%) levels. In Table 1, this has been presented ordinally with higher means indicative of higher levels of education.

Moreover, women with PCOS, relative to those without PCOS, reported greater emotion dysregulation and rumination (large effect sizes), as well as higher rates of NSSI, recent suicidal ideation and future suicidal intention (small to medium effect sizes) (see Table 1). Moreover, Chi-Square analyses revealed that the presence of diagnoses of both anxiety (X^2^ (1, *n* = 418) = 3.95, *p* = 0.047, V = 0.183) and depression (X^2^ (1, *n* = 418) = 4.34, *p* = 0.037, V = 0.192) was significantly more likely in women with, relative to those without, PCOS (small effect sizes). 

### 3.3. Correlations and Serial Mediation Analysis

Data were analysed in IBM SPSS statistics (v.26) using the PROCESS (v.3.4) macro [39]. As evidenced in Table 2, a diagnosis of PCOS was associated with elevated scores on measures of emotion dysregulation, rumination, NSSI and both recent and future suicidal ideation. Moreover, emotion dysregulation was positively associated with rumination, with both variables positively associated with NSSI and suicidal ideation. Recent suicidal ideation and NSSI were both positively associated with one another as well as future suicidal intention. Typically, these correlations ranged from weak to moderate in nature, suggesting not only a need for replication of these findings, but also further analysis of potential covariates explaining the variation in these relationships. 

**Figure 1:**
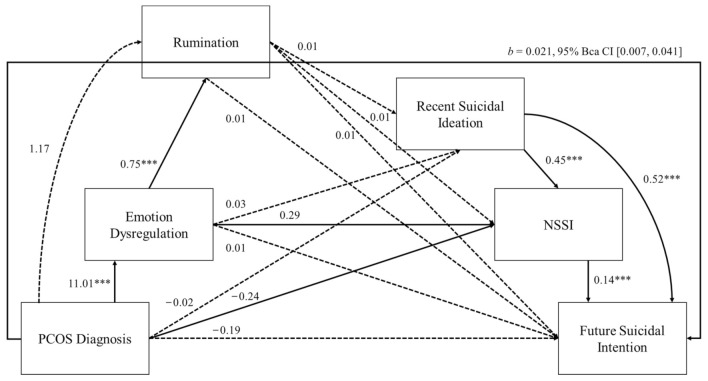
Full series mediation model. Notes. *** *p* < 0.001; regression coefficients are unstandardized; solid lines = significant paths; dotted lines = non-significant paths. Model controls for variation in age, education and ethnicity.

The primary hypothesis that emotion dysregulation, rumination, recent ideation and NSSI would mediate the relationship between diagnosis of PCOS and future suicidal intention was tested using serial mediation analysis (least squares path analysis), controlling for age, education and ethnicity as covariates. All predictor (PCOS diagnosis) and mediator (emotion dysregulation, rumination, recent suicidal ideation, NSSI) variables showed a linear relationship with future suicidal intention, and there was no evidence of multicollinearity. As expected, the distributions of the three indicators of self-injury and suicidal ideation deviated from normality, with positive skew and kurtosis (see Table 2). However, and as per Fadoir et al. [24], the serial mediation analysis uses a bootstrapping procedure whereby normal sampling distributions are not required, and so deviations in analysis plans were not required [40]. This generated 95% bias-corrected bootstrapped confidence intervals of indirect effects (5000 bootstrap samples), which were deemed to be statistically significant if the confidence intervals did not span zero. As positioned in Figure 1, paths leading from PCOS to emotion dysregulation, emotion dysregulation to rumination, recent suicidal ideations to NSSI and NSSI to future suicidal intention were all statistically significant (*p* < 0.001). The path from rumination to recent suicidal ideations was non-significant (*p* = 0.178). The total effect of diagnosis of PCOS on future suicidal intention was non-significant; *b* = 0.23, *p* = 0.139. The specific indirect effect of the serial mediation model with paths from PCOS to emotion dysregulation, to recent suicidal ideation, to NSSI and to future suicidal intention was statistically significant; *b* = 0.021, 95% BCa CI [0.007, 0.041]. The direct effect of diagnosis of PCOS on future suicidal intention (path c’) was non-significant, *b* = −0.190, *p* = 0.098, suggesting full mediation of this relationship. 

## 4. Discussion

The preliminary evidence presented within this report suggests that the presence of a diagnosis of PCOS is associated with a greater prevalence of recent suicidal ideation, non-suicidal self-injury and future suicidal intention, as well as greater self-reported scores on measures of rumination and deviant emotion regulation strategy use. Moreover, it suggests that the positive relationship between a PCOS diagnosis and future suicidal intention is explained through the indirect pathway of increased emotion dysregulation, recent suicidal ideation and NSSI. Taken together, our hypotheses were overall supported and will be discussed below in the context of previous literature and policy. 

The first advancement of knowledge which can be taken from this research builds on emerging yet limited evidence pertaining to suicide-related emotion regulation in women with PCOS. Specifically, women with, relative to women without, a diagnosis of PCOS in our sample were seemingly at a greater risk of exhibiting more deviant emotion regulation strategies, as well as more prevalent suicide-related cognitions (i.e., rumination, suicidal ideation and intent) and behaviour (i.e., NSSI). Previously, women with, relative to those without, a diagnosis of PCOS have been shown to exhibit a preference for dysfunctional emotional regulation strategy use and rumination [4,5]. Moreover, not only has this emotion dysregulation been evidenced on a behavioural level, but it has also been evidenced on a biological level via atypical variation in prefrontal cortex functioning [4]. Although our index of emotion dysregulation was self-reported and not a behavioural measure, the DERS [35] is a well-validated assessment tool which has recently been used to assess emotion dysregulation in predictions of suicide intent [24].

In accordance with the above finding, we also found women with, relative to women without, a diagnosis of PCOS reported a greater frequency of suicidal ideation and NSSI, as well as a higher future suicidal intent. These findings support existing evidence from both quantitative and qualitative perspectives which report associations between PCOS and an increased risk of hospitalisation for self-harm (in addition to stress, anxiety, depression; [13]) and experiences of PCOS-driven suicidal ideation and engagement in self-harm behaviour [14,17]. More broadly, this contributes to the evidence pool from Sweden whereby suicide attempts were found to be seven times more common for women with PCOS compared to age-matched controls [18], and where women with PCOS were found to be 40% more likely to attempt suicide than their non-PCOS counterparts [19]. Taken together, our observed relationships between PCOS diagnoses and suicide-related cognitions and behaviours are unsurprising; however, they further highlight a need for greater understanding and intervention in this demographic at risk of suicide and self-harm. 

The second advancement of knowledge from this research is situated within the use of serial mediation analysis to test the ideation-to-action framework [21] in the context of PCOS as a pre-cursor to future suicide intent. This framework was most recently explored in the context of the relationship between psychopathy and suicidal intent [24] and argues that to attempt suicide, one must both hold intent to end their own life as well as the means to do so [22]. Specifically, this research used the model proposed and validated in Fadoir et al. [24] and found that the relationship between having a diagnosis of PCOS and future suicidal intent was mediated through the indirect pathway of elevated emotion dysregulation, recent suicidal ideation and NSSI. Simply put, it is plausible that the increased use of dysfunctional emotion regulation strategies used by women with PCOS (detailed above; [3]) may contribute to a greater propensity to engage in NSSI as a means of either managing extreme emotions or serving as a distraction or call for help [29,30]. Moreover, it is then likely that self-harm, evidence of one’s ability to take the necessary steps of engaging in behaviours which could lead to suicide completion [32], as well as overcoming fears stemming from suicidal ideation, such as those involved in the contemplation, planning and preparation of suicide [23], might better enable a woman with PCOS to complete suicide. With deliberate self-harm thought to be the strongest risk factor for future suicide [20], recognising associated risks to and predictors of women with PCOS engaging in such behaviour is vital in the movement to safeguard this population. 

One unexpected finding of this research, however, is that unlike the results reported in Fadoir et al. [24], our model did not find the variable rumination to be a significant contributor to this mediation pathway. Nevertheless, rumination, a maladaptive emotion regulation strategy that involves the repetitive engagement of negative thoughts, was statistically and positively associated with both the presence of a diagnosis of PCOS and future suicidal intent at baseline, which were expected results. Taken together, although perhaps not a core mechanism underpinning the processes relating PCOS and suicidal intent in the proposed model, in the context of rumination being previously suggested to underpin suicidal ideation elsewhere as well [26,27], future investigation examining the experiences of rumination in PCOS should not be discounted. 

Results of this investigation are discussed considering five limitations. First, this research is cross-sectional in nature, with data only recorded during a single timepoint. Even though this in no way detracts from the relevance and importance of the findings reported here, it does neglect the potential confounding that one’s risk for attempting suicide fluctuates over time [41]. As such, it is possible that the mediation model presented in this investigation might be further moderated by variation in time and events (both positive and negative) which occur during said timeframe. Second, we relied on women with PCOS self-reporting their diagnosis over recruiting directly from clinical samples and did not ask them to verify their diagnosis. We contend that we have no reason to believe any meaningful variation in our data would be gleaned by potential deception, of which there is no direct benefit, and that this recruitment method overcomes limitations of recruiting directly from health services, such as time, expense and resources. Regardless, it would be useful to verify these results within a clinical sample. It is also possible that women with severe psychological symptoms may not have the opportunity to take part in this (and other) studies due to their ongoing health conditions, and as such, future research should seek to work alongside PCOS patients to identify how best to reach this important group. Third, we acknowledge other factors that are likely to predict suicidal behaviour, such as the presence of substance use disorder, time since PCOS diagnosis, fertility, undergoing PCOS treatment, past history of abuse, anxiety disorders, marital status and socioeconomic factors. Though our intended sample size restricted our ability to incorporate these co-variates into our models in this study, we call for a need for future research to delineate the impact of these factors on the pathways between PCOS diagnosis and future suicidal intention. Fourth, we did not age-match control our two groups. Although this is not explicitly limiting for our path model, it is a practice which should be considered in future work in this field to better allocate variance in NSSI, suicidal ideation and emotion dysregulation to PCOS specifically. Finally, we also acknowledge the potential role of comorbidities not measured in the current sample, such as cancer, which would likely impact measures of NSSI and future suicidal ideation, among others. 

## 5. Conclusions

The study findings present several key directions for future research. Initially, replication both clinically and internationally is needed to establish the prevalence of suicide ideation and self-harm behaviours, alongside the role of emotion regulation in mediating these behaviours, in these samples. Moreover, there is benefit to be had from a qualitative exploration into better understanding perceptions and cognitions underlying suicide ideation and self-harm behaviours in women with PCOS. In addition, further exploration of emotion regulation and its manifestations in women with PCOS is needed, alongside development, testing and adaption of established emotion regulation strategies; for example, nature connectedness [42] and mindfulness [43] in women with PCOS specifically. 

Taken together, this research highlights value in using the ideation-to-action framework to better understand suicide in women with PCOS and further indicates an increased propensity for such women to engage in greater NSSI and suicidal ideation, as well as to report future suicidal intention. As such, we suggest that the international guideline recommendations (ESHRE) for screening depression and anxiety in women newly diagnosed with PCOS [12], whilst a positive step forward, are not enough. Our findings indicate that women with PCOS, internationally, should also be screened for suicide intention and self-harm behaviours, not only when newly diagnosed but across their lifespan. 

## Figures and Tables

**Table 1:** Comparisons of variables of interest between women with (*n* = 113) and without (*n* = 305) a diagnosis of PCOS.

	M_PCOS−_ (SD)	M_PCOS+_ (SD)	*t*	*p*	95% CI	*d*
Age	33.65 (11.38)	31.01 (6.33)	2.33	0.02	(0.415, 4.859)	0.29
Education	2.73 (0.90)	2.51 (0.97)	2.196	0.03	(0.023, 4.19)	0.24
Emotion Dysregulation	37.50 (15.54)	50.18 (16.77	−7.25	<0.001	(−16.120, −9.244)	0.78
Rumination	46.38 (14.28)	56.98 (13.94)	−6.78	<0.001	(−13.670, −7.527)	0.75
Recent suicidal ideation	0.65 (1.04)	1.15 (1.16)	−4.21	<0.001	(−0.730, −0.266)	0.45
NSSI	1.19 (1.69)	1.57 (1.70)	−2.04	0.04	(−0.746, −0.013)	0.22
Future suicidal intention	1.00 (1.19)	1.33 (1.45)	−2.33	0.02	(−0.598, −0.051)	0.25

**Table 2:** Descriptive statistics, reliability, and bivariate correlations (*n* = 418).

Variable	1	2	3	4	5	6
1. PCOS	-	0.335 ***	0.316 ***	0.202 ***	0.099 *	0.113 *
2. Emotion Dysregulation	-	-	0.853 ***	0.583 ***	0.475 ***	0.500 ***
3. Rumination	-	-	-	0.527 ***	0.418 ***	0.471 ***
4. Recent suicidal ideation	-	-		-	0.459 ***	0.624 ***
5. NSSI	-	-	-	-	-	0.457 ***
6. Future suicidal intention	-	-	-	-	-	-
M(SD)	-	40.92 (16.84)	49.25 (14.93)	0.79 (1.09)	1.29 (1.70)	1.09 (1.27)
*z*-Skewness	-	3.31	1.94	11.11	11.79	9.59
*z*-Kurtosis	-	−3.70	−2.65	3.68	5.94	6.22
*a*	-	0.95	0.96	-	-	-

Notes. * *p* < 0.05, *** *p* < 0.001.

## Data Availability

Raw data can be viewed here: https://osf.io/28p7b/?view_only=0400dccde50c4f02b767eca50027caa3 (accessed on 4 May 2022).
